# Is It Safe to Switch From Intravenous Immunoglobulin to Subcutaneous Immunoglobulin in Patients With Common Variable Immunodeficiency and Autoimmune Thrombocytopenia?

**DOI:** 10.3389/fimmu.2018.01656

**Published:** 2018-07-19

**Authors:** Philipp Scheuerlein, Larissa Pietsch, Nadezhda Camacho-Ordonez, Veronika Reiser, Smita Patel, Siobhan O. Burns, Klaus Warnatz, Bodo Grimbacher

**Affiliations:** ^1^Center for Chronic Immunodeficiency, University Hospital, Medical Faculty, Albert-Ludwigs University, Freiburg, Germany; ^2^Faculty of Biology, Albert-Ludwigs University, Freiburg, Germany; ^3^Institute for Medical Biometry and Statistics, Medical Faculty, Albert-Ludwigs University, Freiburg, Germany; ^4^Department of Clinical Immunology, John Radcliffe Hospital, University of Oxford, Oxford, United Kingdom; ^5^Department of Immunology, Royal Free Hospital, Institute of Immunity and Transplantation, University College London, London, United Kingdom

**Keywords:** common variable immunodeficiency, autoimmune thrombocytopenia, intravenous immunoglobulin, subcutaneous immunoglobulin, immunoglobulin replacement therapy

## Abstract

**Background:**

A significant amount of common variable immunodeficiency (CVID) patients manifest with autoimmunity. Particularly, autoimmune thrombocytopenia (AITP) is commonly seen. Intravenous immunoglobulins (IVIG) are an established treatment option for both, CVID and AITP. Nonetheless, due to fewer systemic side effects, immunoglobulins are increasingly applied subcutaneously (SCIG).

**Objective:**

To compare the efficacy and safety of IVIG and SCIG treatment in patients with both CVID and clinical relevant thrombocytopenia in the prevention of AITP bouts.

**Methods:**

Patients with both CVID and AITP were enrolled at the Centre for Chronic Immunodeficiency in Freiburg, Germany and at the Royal Free Hospital, London, UK. Clinical and laboratory features of patients were collected and analyzed.

**Results:**

This retrospective study recruited 61 adult patients between 19 and 71 years of age who had a diagnosis of CVID and at least one bout of thrombocytopenia defined as a platelet count of <50,000/μl if bleeding episodes occurred, or a platelet count of <20,000/μl without bleeding. Thirty patients received immunoglobulin through IVIG, and 31 patients were on SCIG replacement. One patient of the IVIG-group was excluded, because of a diffuse large B-cell lymphoma. We did not find a higher occurrence of thrombocytopenic events in CVID patients who received SCIG, compared to CVID patients who had IVIG, but we identified a low IgG through level as a risk factor for AITP bouts.

**Conclusion:**

SCIG is at least as safe as IVIG for patients with CVID and concomitant AITP. However, an IgG through level under 7 g/l is a key factor for the development of AITP.

## Introduction

Common variable immunodeficiency (CVID) is the most prevalent symptomatic primary immunodeficiency (1). It is characterized by hypogammaglobulinemia and an impaired antibody response leading to recurrent and chronic infections (2). On the other hand, a significant amount of patients manifest with autoimmunity. Specifically, autoimmune thrombocytopenia (AITP) is commonly seen in patients with CVID with an incidence of up to 14% (3, 4). Intravenous immunoglobulin (IVIG) is being used as a replacement therapy [immunoglobulin replacement therapy (IGRT)] for CVID. However, IVIG can also be used at high doses for the treatment of AITP. It is thought that maintenance IGRT might reduce or even prevent recurrent bouts of AITP. Currently, immunoglobulins are increasingly applied subcutaneously in patients with CVID, as there are fewer systemic side effects compared to intravenous applications and it seems to improve the patients’ quality of life (5, 6).

Therefore, the objective of this study was to answer the question: Is it safe to switch patients with CVID and AITP from IVIG to subcutaneous immunoglobulin (SCIG) treatment, with respect to the prevention of clinically relevant thrombocytopenia?

## Materials and Methods

Patients with both CVID, based on the European Society for Immunodeficiencies criteria, and clinically relevant AITP were included in this cohort study. This retrospective study covered 5 years of chart reviews between 2011 and 2015. Patients were recruited at the Center for Chronic Immunodeficiency in Freiburg, Germany and at the Royal Free Hospital, London, UK. Information on immunological findings, clinical manifestations, and immunoglobulin replacement therapy was collected.

The primary study endpoint was a severe thrombocytopenic event, defined as a platelet count of <50,000/μl if bleeding episodes occurred, or a platelet count of <20,000/μl without bleeding. To investigate the incidence of thrombocytopenic events in each group (IVIG vs. SCIG), we retrospectively reviewed participants’ platelet counts over a period of 5 years.

We estimated the frequency of thrombocytopenic events by summing up all 6-month periods, in which patients had at least one documented thrombocytopenic event, since the majority of participants had a routine blood draw every 6 months and additionally when bleeding events (including petechial) occurred.

Differences between groups were analyzed using the non-parametric Chi-square test and Mann–Whitney *U*-test. Results are illustrated using bar charts or box plot diagrams; with boxes representing the lower quartile, the median and the upper quartile, while the whiskers show the 10th and 90th percentiles. A Kaplan–Meier analysis was performed to evaluate the occurrence of primary study endpoint. The Kaplan–Meier curves were compared using log-rank test. Data were analyzed using GraphPad Prism version 7.01 (GraphPad Software, USA). *p* < 0.05 was considered significant.

Written consent was obtained of all patients. This retrospective study was performed in accordance with the ethical standards of the Helsinki declaration and was approved by the institutional review boards of the two hospitals. Ethics protocol No. 295/13 from the University Hospital of the Albert Ludwigs University, Freiburg; and No. 04/Q0501/119 for the Royal Free Hospital, University College London, Institute of Immunity and Transplantation, London, UK.

## Results

This retrospective cohort study recruited 61 adult patients between 19 and 71 years of age who had a diagnosis of CVID and at least one event of clinical significant thrombocytopenia at any time during their medical history. Forty-two patients were enrolled at the Center for Chronic Immunodeficiency in Freiburg, Germany, and 19 patients were recruited at the Royal Free Hospital in London, UK. All participants were on a stable dose of IgG replacement (there was no more variation than 10%), with a median of 477.5 mg/kg/month (range: 232–942), and a target trough level of >7g/l. Thirty patients received immunoglobulin through the intravenous route (IVIG), and 31 patients were on subcutaneous immunoglobulin (SCIG) replacement.

Two patients changed from IVIG to SCIG during the observation period. They were documented in the IVIG cohort until the date of switching and included in the SCIG cohort 3 months after switching from IVIG to SCIG. No patient switched from SCIG to IVIG. Participants had platelet counts and IgG trough levels determined at least every 6-month, and additionally during bleeding episodes (Table 1). Trough levels used for the analysis represent the average level among measurements.

**Table 1:** Patient demographics and disease characteristics.

ID	Age range	IgG replacement	IgG dose (g/month)	IgG level^a^ (g/L)	Median thrombocytes (Tsd/μl)	Immunomodulation	Other disorders
1	36–40	IV	20	9.83	312	No	Splenomegaly
2	46–50	IV	90	10.9	189	Rituximab	Splenomegaly, AIN
3	31–35	IV	120	10.5	151	Rituximab	Splenomegaly
4	51–55	IV	25	**15.8**	**25**	No	Splenectomy, lymphoma
5	21–25	SC	25	**3.9**	**25**	No	Splenomegaly
6	21–25	IV	20	4.3	211	Steroids	Splenomegaly, AIN
7	51–55	IV	20	8.2	134	Steroids	Splenomegaly
8	51–55	SC	20	8.8	200	No	Splenomegaly
9	21–25	IV	20	**5.7**	**25.6**	Steroids	Splenectomy
10	51–55	SC	25	**8.8**	**45.1**	Steroids	None
11	36–40	IV	20	8	145	No	Splenectomy
12	41–45	SC	32	6.1	250	Steroids	Splenomegaly
13	51–55	SC	45	10	107	Steroids	Splenomegaly
14	51–55	SC	33	7.0	140	Steroids	Splenomegaly
15	56–60	SC	20	5.1	136	No	Splenomegaly
16	51–55	SC	64	8.7	257	No	Splenomegaly
17	41–45	SC	30	9.2	158	Steroids	Splenomegaly
18	36–40	SC	25	9.8	181	No	Psoriasis
19	26–30	SC	28	**7.5**	**28**	Steroids	Splenomegaly
20	51–55	IV	20	**9.8**	**20**	Methotrexate, steroids	Splenomegaly, rheumatoid arthritis, antiphospholipid syndrome
21	51–55	SC	50	9.4	147	Rituximab	Splenomegaly
22	41–45	SC	38	**7.8**	**38**	Steroids	Splenomegaly
23	26–30	IV	45	15.6	97	Steroids	Splenomegaly
24	46–50	SC	51	10.9	60	Steroids	Splenomegaly
25	46–50	SC	30	6.1	300	No	Splenectomy
26	41–45	SC	64	8.4	75	No	Splenomegaly
27	61–65	SC	20	**8.7**	**20**	No	Splenomegaly
28	51–55	IV	38	**11.2**	**38**	No	Splenomegaly
29	41–45	SC	38	9.4	114	Steroids, ciclosporin	Splenomegaly, autoimmune enteropathy, vitiligo
30	21–25	SC	35	8.1	205	No	Splenomegaly
31	36–40	SC	20	**8.7**	**12.8**	No	None
32	21–25	SC	40	7.0	126	No	Splenomegaly
33	31–35	IV	20	9.6	175	No	Splenectomy
34	61–65	SC	36	**9.5**	**36**	Rituximab	Splenomegaly
35	41–45	SC	40	12.0	170	Steroids	Splenomegaly
36	56–60	IV	28	9.8	177	No	Celiac disease
37	36–40	SC	53	7.1	84	Rituximab	Splenomegaly
38	21–25	IV	20	**5.9**	**80**	No	Splenectomy
39	26–30	IV	20	10.6	152	No	Splenomegaly
40	31–35	IV	20	**8.7**	**20**	No	Splenomegaly
41	66–70	IV	25	10.8	160	No	Splenomegaly
42	26–30	SC	20	**6.4**	**20**	No	Splenomegaly
43	36–40	SC	30	12.7	220	Rituximab	Splenectomy
44	61–65	IV	40	9.8	305	No	None
45	46–50	IV	30	8.4	220	No	None
46	51–55	IV	50	8.8	146	Rituximab	AIHA
47	31–35	SC	40	8.2	175	No	None
48	31–35	IV	30	13.5	158	Rituximab	AIN
49	21–25	IV	35	8.0	442	Rituximab	Splenectomy
50	61–65	IV	40	14.8	253	No	Splenectomy
51	36–40	SC	30	9.4	182	No	None
52	71–75	IV	30	**13**	**11.2**	Rituximab	Splenectomy
53	36–40	IV	35	5.2	141	No	None
54	46–50	IV	40	11.2	132	Rituximab	None
55	51–55	IV	30	**7.9**	**8.6**	No	None
56	51–55	SC	20	11.2	308	No	None
57	31–35	IV	30	16.3	169	No	None
58	41–45	IV	30	7.9	131	No	None
59	36–40	SC	30	8.6	233	No	Splenectomy
60	16–20	SC	30	10.2	150	No	None
61	56–60	IV	25	10.7	253	No	Splenectomy

*IV, intravenous; SC, subcutaneous; AIHA, autoimmune hemolytic anemia; AIN, autoimmune neutropenia*.

*^a^IgG levels under replacement therapy*.

*Bold numbers indicate patients with autoimmune thrombocytopenia*.

Reviewing the patients’platelet counts and patient-charts over the last 5 years, we counted eight patients in the SCIG-group, and eight patients in the IVIG-group, which were affected by thrombocytopenic events. However, one patient of the IVIG-group had to be excluded, since his thrombocytopenic events were directly related to chemotherapy he had been receiving because of a diffuse large B-cell lymphoma. All other thrombocytopenic events were classified as AITP.

Patients with AITP under IVIG substitution had infusions scheduled every four weeks; five of them had about of AITP during the third week after infusion. SCIG patients with AITP had their infusions scheduled as follows: six patients had one infusion per week; one patient had injections three times a week, and one individual had infusions every 10 days.

**Figure 1:**
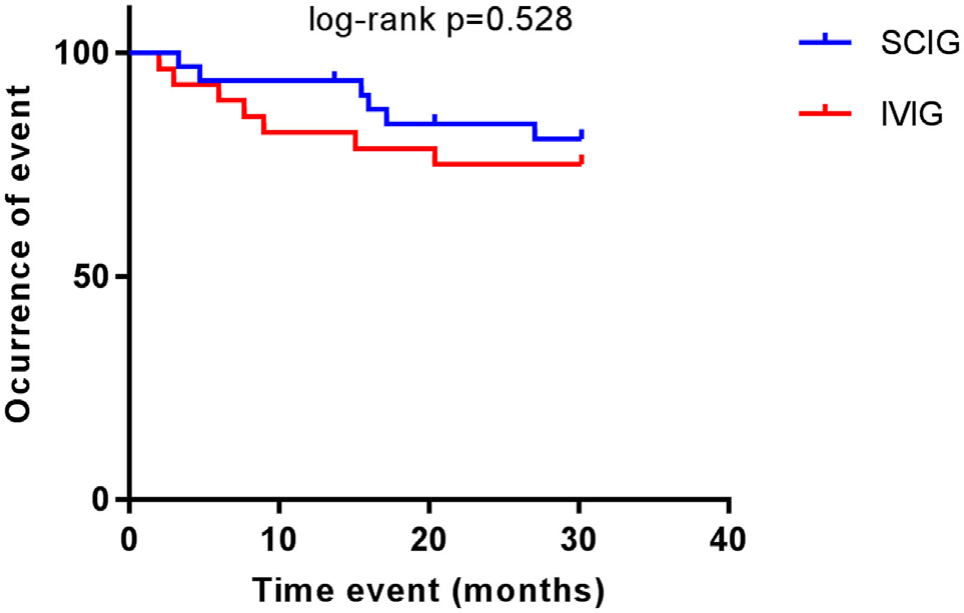
Common variable immunodeficiency (CVID) patients with autoimmune thrombocytopenia on replacement therapy. Kaplan–Meier curves showing the occurrence of thrombocytopenic events in patients receiving subcutaneous immunoglobulin (SCIG) and intravenous immunoglobulin (IVIG). Log-rank test was used to analyze difference between groups. No differences were observed (*p* = 0.528).

**Figure 2:**
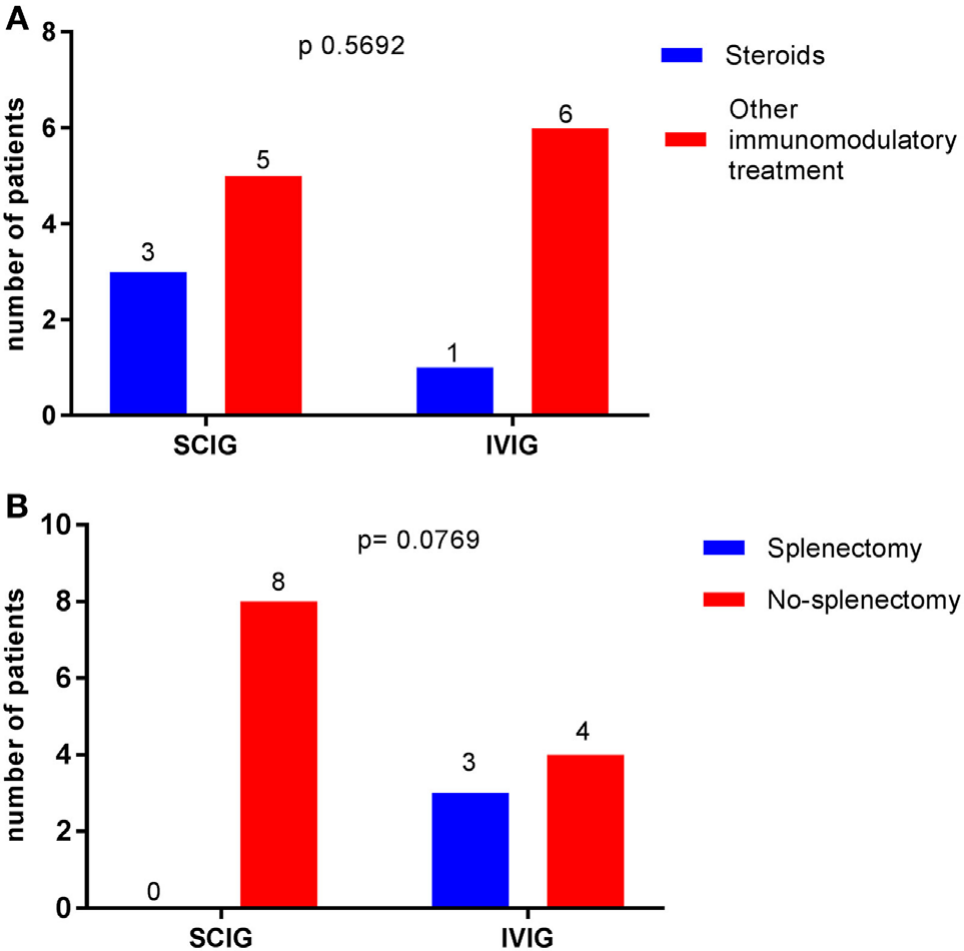
Comparison between immunoglobulin replacement therapy (IGRT) and potential confounding factors in the occurrence of autoimmune thrombocytopenia (AITP) bouts. Data are expressed as the number of patients with AITP bouts receiving IGRT (the total is 14). Chi-squared was used. **(A)** No difference was observed when comparing patients receiving steroids or other immunomodulatory treatment *p* = 0.5692. **(B)** Status of splenectomy made no difference in the occurrence of thrombocytopenic events *p* = 0.0769.

There were no statistically significant correlations between gender (*p* = 0.3248) and thrombocytopenic events. When comparing Ig replacement, there were no significant differences in the occurrence of thrombocytopenia between the IVIG and SCIG-group (Figure 1). We, therefore, concluded that CVID patients who received subcutaneously immunoglobulin treatment were not more susceptible to recurrent thrombocytopenic events than CVID patients on IVIG substitution. Moreover, we excluded steroid treatment and splenectomy as confounding factors as we did not observe a significant difference when comparing AITP patients on IVIG or SCIG (Figures 2A,B).

**Figure 3:**
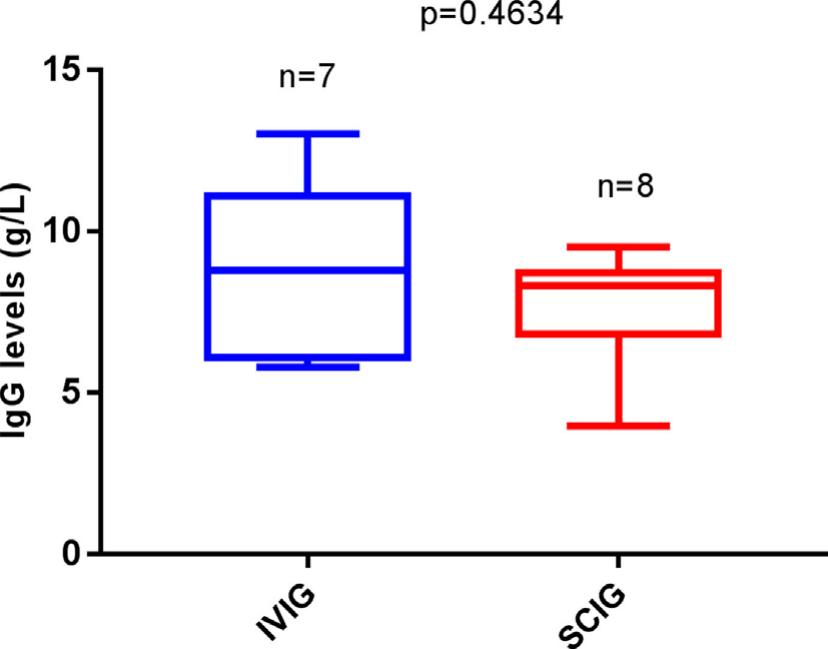
Replacement therapy among autoimmune thrombocytopenia (AITP) patients related to IgG through levels. Number of patients with AITP bouts under intravenous immunoglobulin (IVIG) (*n* = 7) and under SCIG (*n* = 8). Box plot diagrams represent the lower quartile, the median and the upper quartile, while the whiskers show the 10th and 90th percentiles. Differences were compared by the Mann–Whitney *U*-test. No differences were observed, *p* = 0.4634. *p* is considered significant when < 0.05.

**Figure 4:**
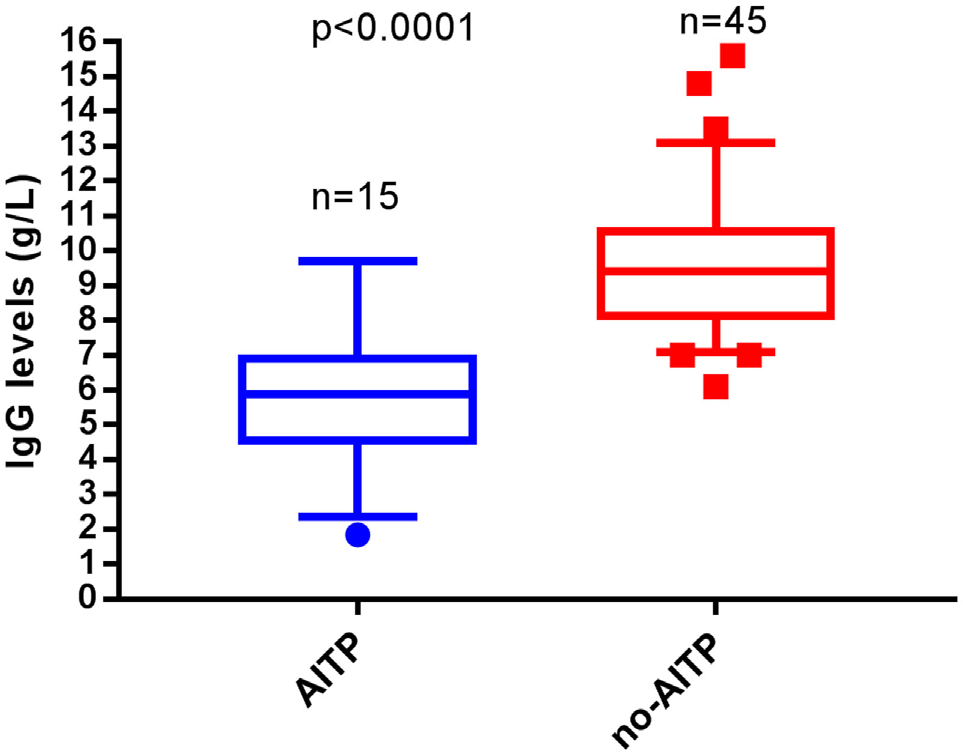
Occurrence of autoimmune thrombocytopenia (AITP) related to IgG through levels. Patients with AITP (*n* = 15) and those without bouts (*n* = 45) are depicted in box plot diagrams. Box plots show the lower quartile, the median and the upper quartile, while the whiskers show the 10th and 90th percentiles. A significant difference was observed among patients with through levels under 7 g/l. From the AITP group 12 patients (80%) had through levels below this cut point, while from the non-AITP group 44 patients (97.7%) where above 7 g/l. Group comparisons were performed by the Mann–Whitney *U* test. *p* is considered significant when <0.05.

Comparing IgG trough levels, we again found no difference between patients in the IVIG-group and the SCIG-group (*p* = 0.4634) (Figure 3). But those who were affected by a thrombocytopenic event had a higher relative frequency of IgG trough-level under 7 g/l in serum compared to patients without a thrombocytopenic event (OR 0.75, 95% CI 0.12–7.03; *p* < 0.0001) (Figure 4). This indicates that a low IgG level is a key factor for the development of thrombocytopenia. In addition, there was no patient affected by autoimmune hemolytic anemia or autoimmune neutropenia during the 5-year observation period.

## Discussion

Given that IGRT is increasingly applied subcutaneously; we expected that this study could help to understand better the role of available options of IGRT in patients with both AITP and CVID in a clinical setting. The German Registry for PID found out that 73% of patients with PID are receiving SCIG and 27% are under IVIG replacement (7).

In this cohort study, the groups differed at baseline in participant characteristics: gender, splenectomy status, immunomodulation, and replacement therapy. The comparability of the groups was questioned by counting the occurrence of influencing factors. We found no statistically significant differences related to gender. Therapeutic options for AITP include steroids, immunosuppressant, and splenectomy (8, 9). However, none of these factors seem to have influenced the IGRT administration route.

Antibodies to thrombocytes were not tested for all patients; the measurement of platelet-associated IgG for the diagnosis of ITP is published to have a sensitivity around 90%, but its specificity is only 27%; hence, the positive predictive value is only <50% and its diagnostic value is poor. While the measurement of specific platelet glycoprotein antibodies has higher specificity (78–92%), its diagnostic value is limited by low sensitivity (49–66%) with a positive predictive value of 80–83% (10). We differentiated hypersplenism from AITP according to the clinical observation of the platelets kinetics: in AITP the drop of platelets is quick, while hypersplenism develops more slowly in CVID and usually leads to a gradual reduction in platelet counts.

When comparing IVIG vs. SCIG replacement with the occurrence of AITP events, we did not find a different incidence of thrombocytopenic events between groups. Although our sample size is limited, our data do not give a signal that SCIG is less safe than IVIG for patients with CVID and concomitant autoimmune cytopenia.

It has been established that target through serum IgG, achieved by either intravenous or subcutaneous route varies and the goal is to prevent infections (1, 11).

Trough levels have not been previously related to thrombocytopenic events. Our study suggests IgG trough-levels under 7 g/l point to be a key factor for development of thrombocytopenia.

This study is limited by its statistical power and its retrospective design. Based on statistical tests we analyzed differences between groups and, therefore, only controlled for type I errors. Due to the limited sample size there is possibly a high risk of type II errors, for which our tests did not control. Hence, further investigations are necessary. This should be addressed in the coming years by collecting prospective data on the above identified 60 patients.

In summary, we did not find a higher occurrence of thrombocytopenic events in CVID patients who received SCIG, compared to CVID patients who had an intravenous application of IgG, but we identified a low IgG through level as a risk factor for ITP bouts.

## Ethics Statement

This retrospective study was performed in accordance with the ethical standards of the Helsinki declaration and was approved by the institutional review boards of the two hospitals. Ethics protocol No. 295/13 from the University Hospital of the Albert Ludwings University, Freiburg; and No. 04/Q0501/119 for the Royal Free Hospital, University College London, Institute of Immunity and Transplantation, London, UK.

## Author Contributions

PS wrote the proposal of the work, acquisition of data, organized the database, and wrote first draft of the manuscript; LP performed acquisition of data and organized the London patient’s database; NC wrote sections of the manuscript, analyzed, and interpreted of data; VR performed statistical analyses. SP performed acquisition of data, provided, and cared for study patients; SB provided and cared for study patients; KW revised the work critically for intellectual content, provided and cared for study patients, and wrote sections of the manuscript; BG performed conception and designing of the study, provided and cared for study patients and revised the work critically for intellectual content. All authors contributed to manuscript revision, read, and approved the submitted version.

## Conflict of Interest Statement

The authors declare that the research was conducted in the absence of any commercial or financial relationships that could be construed as a potential conflict of interest.
